# Whole Genome Mapping Reveals Novel Genes and Pathways Involved in Milk Production Under Heat Stress in US Holstein Cows

**DOI:** 10.3389/fgene.2019.00928

**Published:** 2019-10-04

**Authors:** Anil Sigdel, Rostam Abdollahi-Arpanahi, Ignacio Aguilar, Francisco Peñagaricano

**Affiliations:** ^1^Department of Animal Sciences, University of Florida, Gainesville, FL, United States; ^2^Instituto Nacional de Investigación Agropecuaria, Canelones, Uruguay; ^3^University of Florida Genetics Institute, University of Florida, Gainesville, FL, United States

**Keywords:** genetic parameters, gene-set analysis, genomic scan, heat-shock proteins, thermotolerance

## Abstract

Heat stress represents a major environmental factor that negatively affects the health and performance of dairy cows, causing huge economic losses to the dairy industry. Identifying and selecting animals that are thermotolerant is an attractive alternative for reducing the negative effects of heat stress on dairy cattle performance. As such, the objectives of the present study were to estimate genetic components of milk yield, fat yield, and protein yield considering heat stress and to perform whole-genome scans and a subsequent gene-set analysis for identifying candidate genes and functional gene-sets implicated in milk production under heat stress conditions. Data consisted of about 254k test-day records from 17,522 Holstein cows. Multi-trait repeatability test day models with random regressions on a function of temperature-humidity index (THI) values were used for genetic analyses. The models included herd-test-day and DIM classes as fixed effects, and general and thermotolerance additive genetic and permanent environmental as random effects. Notably, thermotolerance additive genetic variances for all milk traits increased across parities suggesting that cows become more sensitive to heat stress as they age. In addition, our study revealed negative genetic correlations between general and thermotolerance additive effects, ranging between −0.18 to −0.68 indicating that high producing cows are more susceptible to heat stress. The association analysis identified at least three different genomic regions on BTA5, BTA14, and BTA15 strongly associated with milk production under heat stress conditions. These regions harbor candidate genes, such as *HSF1*, *MAPK8IP1,* and *CDKN1B* that are directly involved in the cellular response to heat stress. Moreover, the gene-set analysis revealed several functional terms related to heat shock proteins, apoptosis, immune response, and oxidative stress, among others. Overall, the genes and pathways identified in this study provide a better understanding of the genetic architecture underlying dairy cow performance under heat stress conditions. Our findings point out novel opportunities for improving thermotolerance in dairy cattle through marker-assisted breeding.

## Introduction

Dairy cattle selection programs have traditionally focused on increasing milk yield and milk solids. For example, average milk yield of US dairy cattle has increased by more than double in the last 50 years, and more than half of that improvement is due to genetic selection (Vanraden, 2004). The intense selection for production, however, has led to increased sensitivity to environmental changes in dairy cattle. Today’s high producing dairy cows tend to be more heat susceptible which negatively impacts health, fertility, and lactation performance (Aguilar et al., 2010b; Nardone et al., 2010; Biffani et al., 2016). Heat stress is an important economic issue in dairy farming. Economic losses due to heat stress are estimated between $897 million to $1,500 million per year for the US dairy sector (St-Pierre et al., 2003). Heat stress is costly for dairy producers, especially in the southern states of the US where climate is subtropical and subject to extended periods of high ambient temperature and humidity. In Florida, dairy cows experience approximately 250 heat stress days during the year and lose about 1,200 kg of milk in the subsequent lactation if they are not cooled during the dry period (Ferreira et al., 2016). Given that heat stress is a costly problem, different approaches such as physical modifications of the environment, and improved nutritional and management practices have been used to alleviate the negative effects of heat stress. However, these practices increase production costs, and in general, they cannot eliminate heat stress completely. One complementary strategy for reducing the effects of heat stress on dairy cattle performance is the identification and subsequent selection of animals that are genetically more thermotolerant. Selective breeding of dairy cattle for thermotolerance is permanent, cumulative, and, therefore, it probably represents the most cost-effective approach for mitigating heat stress effects.

There is growing evidence that there is substantial genetic variation underlying cow response to heat stress, and hence, genetic selection for improved thermotolerance is possible in dairy cattle (Ravagnolo and Misztal, 2000; West, 2003; Nguyen et al., 2017). Some indicator traits, such as rectal temperature, exhibit a sizable genetic component. For instance, Dikmen et al. (2012) estimated heritability of rectal temperature in US Holsteins between 0.13 and 0.17. The magnitude of the heritability estimate suggests that genetic selection for response to heat stress is possible. However, the inclusion of this indicator trait in a national genetic evaluation is both expensive and cumbersome. An alternative methodology to evaluate heat stress is to examine the decline in production per unit increase in temperature-humidity index (THI) as THI increases above a given threshold (Ravagnolo and Misztal, 2000). In this context, a linear regression of a performance trait such as milk yield on environmental variable (THI) is fitted to predict the relationship between production and weather conditions. This model assumes that production is unaffected until a certain threshold level of THI, and above that level, production declines linearly with increasing THI, and the slope can be considered as a measure of susceptibility to heat stress. Genetic variation is associated with the amount of production loss above a certain threshold level. Using this approach, Nguyen et al. (2016) estimated breeding values for heat tolerance in Australian dairy cattle which provides opportunity to breed cows that are more thermotolerant and have lower decline in milk yield during heat stress conditions.

There are few studies that have reported associations between genomic regions and thermotolerance in dairy cattle. For instance, Dikmen et al. (2013) identified a genomic region on BTA24 to be significantly associated with rectal temperature in dairy cows. Olson et al. (2003) reported the *Slick* gene as a major candidate gene influencing hair length and regulating thermotolerance in *Bos taurus* cattle. Recently, Macciotta et al. (2017), using principal component analysis, detected a genomic region on BTA26 associated with milk yield under heat stress conditions. Srikanth et al. (2017) identified genes involved in apoptosis, immune response, and metabolism as major genes implicated in the heat stress response of Holstein bull calves subjected to heat stress conditions for 12 hours. Moreover, several studies have reported heat shock factors as principal molecular chaperones involved in cellular response to heat stress in dairy cattle, including protection from protein aggregation, misfolding, and thermal insults (Collier et al., 2008; Min et al., 2015; Min et al., 2017). On the other hand, there is limited knowledge on individual genes and functional pathways implicated in cow’s ability to produce milk under heat stress conditions. Therefore, the first objective of this study was to estimate genetic components of yield traits across lactations considering heat stress using random regressions as a function of THI values. The second objective of this study was to perform whole-genome scans and a subsequent gene-set enrichment analyses in order to identify genes and gene networks responsible for milk production under heat stress conditions.

## Materials and Methods

### Phenotypic and Genotypic Data

Data consisted of about 254k milk, fat, and protein test-day records on 17,522 Holstein cows calved from 2006 through 2016 on two dairy herds in the state of Florida, United States (**Table 1**). Lactation records were required to have at least 6 test-days and to be between 5 and 305 DIM. Please note that we only worked with datasets and never directly with animals, so ethics approval for the study was not needed. Pedigree was created by tracing the pedigree of cows back to five generations. The pedigree file included 35,006 animals including 3,478 sires.

**Table 1:** Summary statistics of Milk Yield, Fat Yield and Protein Yield by Parity.

	Milk Yield (kg)			Fat Yield (kg × 100)			Protein Yield (kg × 100)		
	Par1	Par2	Par3	Par1	Par2	Par3	Par1	Par2	Par3
No. of cows	15,536	11,453	7,301	15,517	11,446	7,298	15,517	11,445	7,298
Test day records	115,790	84,663	53,762	126,279	92,240	58,771	130,628	95,038	60,698
Mean (SD)	33.03 (6.35)	37.59 (8.65)	39.23 (9.31)	119.89 (27.67)	143.82 (40.95)	151.08 (45.53)	99.55 (18.62)	116.39 (24.52)	119.96 (25.86)

Genotype data for 60,671 single nucleotide polymorphism (SNP) markers were available for 4,753 cows with test-day records and 1,590 sires in the pedigree file. The SNP data were kindly provided by the Cooperative Dairy DNA Repository and the Council on Dairy Cattle Breeding. Those SNP markers that mapped to sex chromosomes, were monomorphic, or had minor allele frequency less than 1% were removed from the genotype data. After data editing, a total of 58,046 SNPs were retained for subsequent genomic analyses.

Weather data were obtained from Florida Automated Weather Network for Alachua County (https://fawn.ifas.ufl.edu/). Hourly THI values were calculated as proposed by Ravagnolo and Misztal (2000) as

THI=(1.8 ⋅ temp+32)−(0.55−0.55 ⋅rh)(1.8 ⋅temp−26)

where *temp* is the temperature in degree Celsius and *rh* is the relative humidity in percentage. Mean daily THI of 3 days prior the test day was assigned to each test-day record as suggested by Bohmanova et al. (2007).

A function of THI, denoted as *f* (*THI*), was created to estimate the reduction in yield traits under heat stress conditions, as follows:

$$f(\text{THI})=\{\begin{array}{ll} 0 & \text{if THl}\leq {\text{THl}}_{\text{thr}}\\ \text{THl}-{\text{THl}}_{\text{thr}} & \text{if THl}>{\text{THl}}_{\text{thr}} \end{array}$$

where the value of *THI_thr_* was set to 68, and thus *f*(*THI*) was equal to *max*(0, *THl − THl_thr_*).

### Statistical Model

Multi-trait repeatability test-day models were used to estimate variance components of milk, fat, and protein yield, considering the first three lactations as different traits:

$$\begin{array}{l} y_{\text{klmn}}={\text{HTD}}_{\text{kl}}+{\text{DIM}}_{m}+a_{\text{nl}}+{\text{pe}}_{\text{nl}}+v_{\text{nl}}[f(\text{THI})]+\\ \;q_{\text{nl}}[f(\text{THI})]+e_{\text{klmn}} \end{array}$$

where *y_klmn_* is the record for the yield trait under consideration, *HTD_kl_* is herd-test-day *k* within parity *l* (*l* = 1, 2, 3), *DIM_m_* is the *m^th^* DIM class with classes defined every 20 days, *a_nl_* is the general random additive genetic effect (intercept) of animal *n* in parity *l*, *pe_nl_* is the general random permanent environmental effect (intercept) of cow *n* in parity *l*, *f(THI)* is a function of THI for herd test day *k*, *v_nl_* is the random regression additive genetic effect (slope) of the yield trait per degree of THI above threshold for the animal *n* in parity *l* (heat tolerance), *q_nl_* is the random regression permanent environmental effect (slope) of thermotolerance of the cow *n* in parity *l*, and *e_klmn_* is the random residual effect.

Let $a=\left[a_{\text{nl}}^{\prime }\;v_{\text{nl}}^{\prime }\right]$ be a vector of random additive genetic effects and $\text{pe}=\left[{{\text{pe}}^{\prime }}_{\text{nl}}\;{q^{\prime }}_{\text{nl}}\right]$ be a vector of random permanent effects for parities *l* = 1 to 3. The (co)variance structure was

$$\text{Var}\left[\begin{array}{c} \text{a}\\ \text{pe}\\ \text{e} \end{array}\right]=\left[\begin{array}{ccc} \text{A}⊗\Phi & 0 & 0\\ 0 & \text{I}⊗\Psi & 0\\ 0 & 0 & \text{I}⊗\text{R} \end{array}\right]$$

where **A** is the numerator relationship matrix, and Φ and ψ are 6x6 (co)variance matrices of random regression coefficients for additive and permanent environment effects respectively (3 traits [3 parities] with 2 parameters representing the intercept and the slope for each trait), **R** is a 3x3 diagonal matrix of residual variances corresponding to each trait, and ⊗ denotes the Kronecker product of matrices.

### Variance Component Estimation

Variance components for yield traits using multi-trait repeatability test-day models were estimated in a Bayesian framework using GIBBS2F90 (version 1.93). Initial values for multiple trait analyses were obtained from Aguilar et al. (2009). Genomic data were not included for variance components estimation. Of a total of 500,000 samples, first 100,000 were discarded as burn-in, and every 100th sample was retained to calculate posterior means and standard deviations of variance components. Convergence diagnostics of Markov chain Monte Carlo sampling output were carried out by visual inspection of trace plots of variance components.

Heritability (*h* ^2^) for each yield trait at heat stress level *f*(*i*) was estimated as

$$h^{2}=\;\frac{\sigma_{a}^{2}+f{\left(i\right)}^{2}\sigma_{v}^{2}+2f(i)\sigma_{\text{av}}\;}{\sigma_{a}^{2}+f{\left(i\right)}^{2}\sigma_{v}^{2}+\;2f(i)\sigma_{\text{av}}+\sigma_{\text{pe}}^{2}+f{\left(i\right)}^{2}\sigma_{q}^{2}+\;2f(i)\sigma_{\text{pq}}+\sigma_{e}^{2}}$$

where $\sigma_{a}^{2}$ the variance of general additive genetic effects; $\sigma_{v}^{2}$ is the variance of thermotolerance additive genetic effects; σ*_av_* is the additive genetic covariance between general and thermotolerance genetic effects; $\sigma_{\text{pe}}^{2}$ is the variance of general environmental permanent effects; $\sigma_{q}^{2}$ is the variance of thermotolerance environmental permanent effects; σ*_av_* is environmental permanent covariance between general and thermotolerance effects, *f*(*i*) is a function of THI, and $\sigma_{e}^{2}$ is the residual variance.

The genetic correlation between general and thermotolerance additive effects was estimated as

$$\text{corr}\;\left[a,\;f(i)v\right]\;=\;\frac{f(i)\sigma_{\text{av}}}{\sqrt{\sigma_{a}^{2}\;f{\left(i\right)}^{2}\sigma_{v}^{2}}}$$

### Gene Mapping

The whole-genome association mapping was performed using single-step genomic BLUP methodology (ssGBLUP). The ssGBLUP model is similar to the classical BLUP model but it replaces the inverse of the pedigree relationship matrix (**A** ^−^ **^1^**) with the inverse of the realized relationship matrix (**H** ^–^ **^1^**) that combines both pedigree and genomic information (Aguilar et al., 2010a). The combined pedigree and genomic relationship matrix **H** ^−^ **^1^** was calculated as

$${\text{H}}^{-1\;}={\text{A}}^{-1}+\;\left[\begin{array}{cc} 0 & 0\\ 0 & {\text{G}}^{-1}-{\text{A}}_{22}^{-1} \end{array}\right]$$

where **G** ^−1^ is the inverse of the genomic relationship matrix and ${\text{A}}_{22}^{-1}$ is the inverse of the pedigree relationship matrix of the animals with genotype information. Here, **G** ^−^ **^1^** has the dimension of 6,343 × 6,343 which includes 4,753 cows with test day records and 1,590 sires in the pedigree. The **A** matrix has a dimension of 35,006 × 35,006 which is based on a five-generation pedigree.

Candidate genomic regions associated with both general additive genetic merit and thermotolerance additive genetic merit for milk production were identified based on the amount of genetic variance explained by 2.0 Mb moving windows of adjacent SNPs. Given the genomic estimated breeding values (GEBVs), the SNP effects can be estimated as $\hat{\text{s}}={\text{DZ}}^{\prime }{\left[{\text{ZDZ}}^{\prime }\right]}^{-1}{\hat{\text{a}}}_{\text{g}}$ where $\hat{\text{s}}$ the vector of SNP marker effects, **D** is a diagonal matrix of weight of SNPs, **Z** is a matrix relating genotypes of each SNP marker to observations, and ${\hat{\text{a}}}_{\text{g}}$ is the vector of GEBVs for genotyped individuals (Wang et al., 2012). The percentage of genetic variance explained by a given 2.0 Mb of moving window of adjacent SNPs was then calculated as

$$\frac{\text{var}(u_{i})}{\sigma_{u}^{2}}\;\times \;100=\;\frac{\text{var}\left(\Sigma_{j=1}^{B}Z_{j}S_{j}\right)}{\sigma_{u}^{2}}\times \;100$$

where *u_i_* is the genetic value of the *i^th^* genomic region under consideration, *B* is the total number of adjacent SNPs within 2.0 Mb region, *Z_j_* is the genotype code of *j^th^* marker, *S_j_* is the marker effect of the *j^th^* SNP within the *i^th^* region. In this study, all the SNPs were equally weighted. All these calculations were performed using POSTGSF90 (version 3.08) of BLUPF90 family of programs (Aguilar et al., 2014).

### Gene-Set Analysis

Whole-genome scans were complemented with gene-set enrichment analyses in order to obtain additional insights regarding biological pathways and molecular mechanisms underlying the genetic variability in milk production under heat stress conditions. As described in detail by Han and Peñagaricano (2016), a gene-set analysis consists basically of three steps: i) the assignment of SNP markers to annotated genes, ii) the assignment of genes to functional gene-sets, and finally iii) the association between each gene-set and the phenotype of interest using Fisher’s exact test.

The UMD 3.1 bovine genome sequence assembly was used for SNP assignment using Bioconductor *R* package biomaRt (version 2.38.0, Durinck et al., 2009). The SNPs were assigned to genes if they were located within the genomic sequence of the gene or at most 15 kb either upstream or downstream the gene. An arbitrary threshold of 5% of the SNP effects distribution (in absolute value) was used to define the set of relevant SNP markers associated with thermotolerance; putative thermotolerant genes were defined as those genes that contained at least one relevant SNP. The Gene ontology (GO) database (Ashburner et al., 2000) and Medical Subject Headings (MeSH) databases (Nelson et al., 2004; Morota et al., 2015) were used to define functional set of genes. Finally, the identification of relevant gene-sets was performed using Fisher’s exact test, a test of proportions based on the cumulative hypergeometric distribution (Peñagaricano et al., 2013).

## Results and Discussion

### Genetic Parameter Estimation

Variance components for lactation performance without heat stress (intercept) and lactation performance under heat stress conditions (slope) for milk yield, fat yield, and protein yield using multi-trait repeatability test day models were estimated using pedigree BLUP (**Table 2**). Relevant genetic parameters include heritability estimates and genetic correlations at heat stress level equal to *f (THI)* = 10 (i.e., 10 units above THI threshold of 68) across the first three parities. We found that both additive genetic variances without heat stress and under heat stress conditions increased across parities. Additive genetic variances for milk yield under heat stress condition increased by 66% from parity one to parity two and 4% from parity two to parity three, suggesting that cows become more sensitive to heat stress as they age. Additive genetic variances under heat stress conditions also increased for milk components in multiparous cows compared to primiparous cows. The increase in additive genetic variances in multiparous cows compared to primiparous cows were in the range of 86% to 167% for fat yield and 16% to 55% for protein yield. Estimates of variance components for general additive genetic merit of milk production traits without heat stress and under heat stress conditions were comparable to those reported by Aguilar et al. (2009) who reported that additive genetic variances without heat stress increased by 25 to 35% from first to second parity and additive genetic variances under heat stress almost doubled from first to second parity. The increase in thermotolerance additive genetic variances across parities was also reported in Australian Dairy cattle (Nguyen et al., 2016). Bernabucci et al. (2014) also reported an increase in heat tolerance additive genetic variances across first three parities in Italian Holstein cows. Overall, the increase in decline of yield traits under heat stress conditions across parities suggest that multiparous cows are more susceptible to heat stress than primiparous cows.

**Table 2:** General $\left(\sigma_{a}^{2}\right)$ and thermotolerance $\left(100\sigma_{v}^{2}\right)$ additive genetic variances, genetic correlations $\left(r_{\left(a,v\right)}^{G}\right)$ and heritability estimates $h_{f(10)}^{2}$ at THI = 78.

Milk Yield (kg)				Fat Yield (kg × 100)^2^			Protein Yield (kg × 100)^2^		
Parameters	Par1	Par2	Par3	Par1	Par2	Par3	Par1	Par2	Par3
$\sigma_{a}^{2}$	9.26	10.03	10.55	119.76	205.77	252.33	55.65	63.80	76.01
$100\sigma_{v}^{2}$	0.94	1.56	1.62	18.19	48.61	33.78	8.57	9.98	13.30
10σ*_av_*	−1.21	−1.17	−2.31	−11.44	−37.90	−63.03	−6.31	−4.58	−12.81
$\sigma_{e}^{2}$	7.31	12.97	15.65	351.15	666.04	840.74	79.92	127.98	154.51
$h_{f(10)}^{2}$ (95% HPD)	0.31 (0.29,0.36)	0.24 (0.21,0.28)	0.17 (0.15,0.20)	0.20 (0.18,0.23)	0.17 (0.14,0.20)	0.13 (0.10,0.15)	0.26 (0.24,0.30)	0.21 (0.18,0.24)	0.18 (0.16,0.21)
$r_{\left(a,v\right)}^{G}$ (95% HPD)	−0.41 (−0.54,−0.28)	−0.30 (−0.46,−0.11)	−0.55 (−0.70,−0.42)	−0.25 (−0.42,−0.06)	−0.38 (−0.56,−0.19)	−0.68 (−0.86,−0.53)	−0.29 (−0.42,−0.15)	−0.18 (−0.38,−0.04)	−0.40 (−0.56,−0.22)
cor_ht_ (par_1_, par_j_)		0.78	0.65		0.46	0.34		0.36	0.55
cor_ht_ (par_2_, par_3_)			0.61			0.38			0.78
cor_gen_ (par_1_, par_j_)		0.82	0.85		0.91	0.95		0.78	0.76
cor_gen_ (par_2_, par_3_)			0.92			0.95			0.96

HPD, highest posterior density; $\sigma_{a}^{2}$, general additive genetic variance; $100\sigma_{v}^{2}$, thermotolerance additive genetic variance at THI = 78; 10σ_av_, additive genetic covariance between general and thermotolerance effect; $r_{\left(a,v\right)}^{G}$, genetic correlation between general and thermotolerance effect; cor_ht_, thermotolerance additive genetic correlation; cor_gen_, general additive genetic correlation.

Heritability estimates for milk production at heat stress level *f (THI)* = 10 were between 0.17 and 0.31 across the first three parities, which is comparable with the heritability estimates reported by Ravagnolo and Misztal (2000). Similarly, heritability estimates for fat yield for the first three lactations were between 13 to 20% whereas heritability estimates for protein yield across the first three lactations were between 18% and 26%. In general, heritability estimates decrease across parity, and this could be because phenotypic variances increase across parity as cows become more sensitive to heat stress in later lactations.

Genetic correlations for milk yield between additive genetic effects without heat stress and under heat stress for the first three parities were all negative, ranging from −0.30 to −0.55. Also, genetic correlations between general and heat tolerance additive effects for milk components were also negative, ranging from −0.18 to −0.68. This is in agreement with the findings of Aguilar et al. (2009) and Ravagnolo and Misztal (2000) in US Holsteins who reported negative genetic correlations between thermotolerance and general merit of milk production. Bernabucci et al. (2014) also reported negative genetic correlations between general and heat tolerance additive effects for yield traits across the first three parities in Italian Holstein.

Overall, our study provides further evidence that production traits are antagonistic to heat tolerance, and that continued selection for milk yield and milk components without consideration for thermotolerance will result in greater susceptibility to heat stress.

### Whole-Genome Mapping

**Figure 1:**
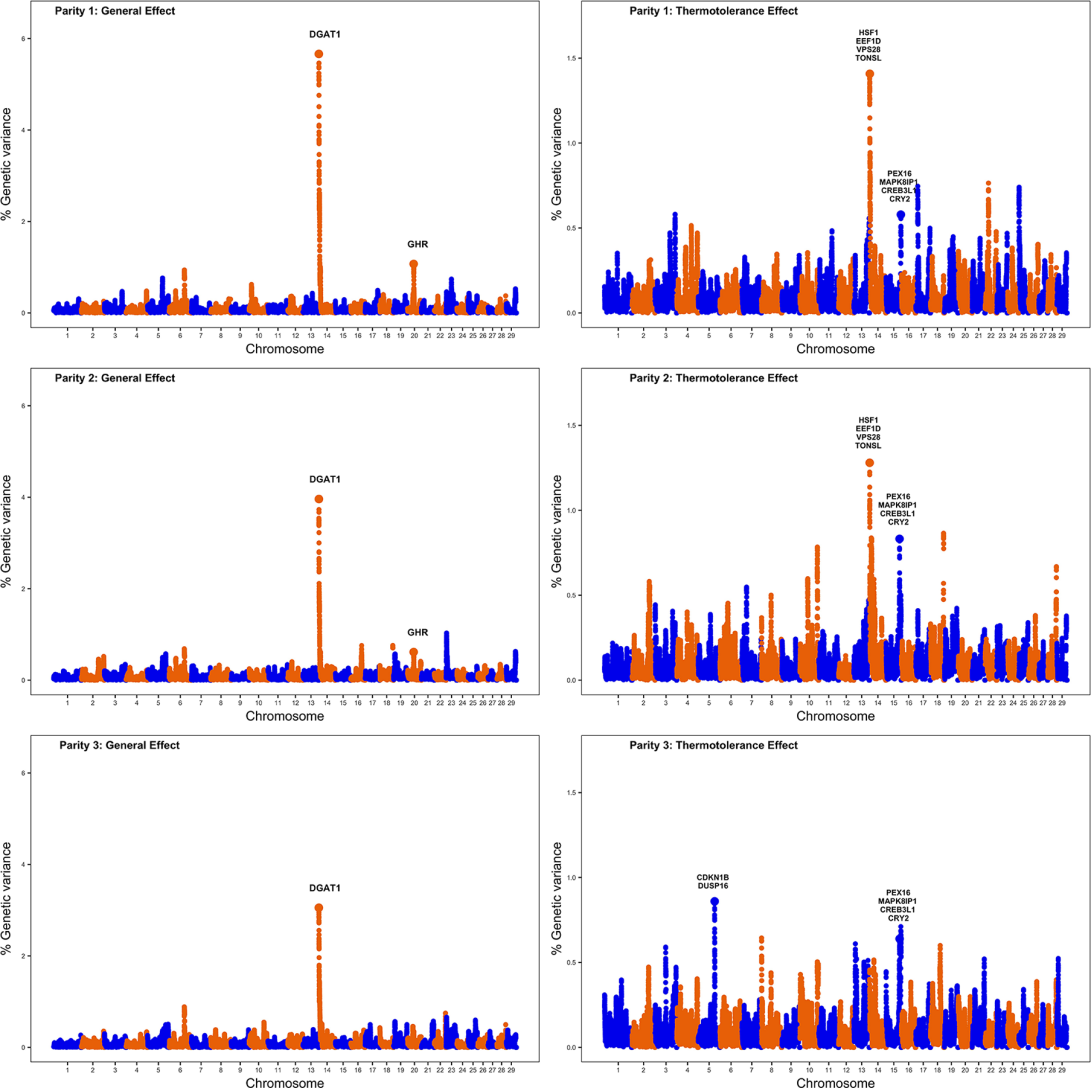
Manhattan plots showing the results of the whole-genome scans for milk production for the first three lactations (numbered vertically as parity 1, parity 2, and parity 3). The left plots highlight genomic regions affecting milk production under thermoneutral conditions (general additive genetic effects), while the right plots highlight genomic regions implicated in milk production under heat stress conditions (thermotolerance additive genetic effects).

Single-step genomic BLUP methodology was utilized to identify genomic regions and putative candidate genes implicated in milk production under thermoneutral and also heat stress conditions. This method combines all the available phenotypic, genotypic, and pedigree information, and fits all the SNPs simultaneously. **Figure 1** displays Manhattan plots for milk production for the three lactations under study; the left plots show genomic regions associated with milk production under thermoneutral conditions while the right plots show genomic regions implicated in milk production under heat stress conditions. The results are presented in terms of the proportion of genetic variance explained by 2.0 Mb SNP windows. As expected, left plots show a clear peak on BTA14 at 1.37–3.37 Mb; this region harbors *DGAT1*, a well-known gene that affects milk yield. Gene *DGAT1* encodes a key enzyme involved in the synthesis of milk triglycerides (Yen et al., 2008). This region on BTA14 that harbors *DGAT1* explained about 5.7%, 4.0%, and 3.0% of genetic variance for milk production across the first three parities.

Another 2.0 Mb SNP window on BTA20 (31.05–33.05 Mb) explained about 1.1% and 0.6% of the additive genetic variance for milk yield in first and second parity, respectively. Notably, this region harbors the gene *GHR*, the growth hormone receptor known to have a major effect on milk yield and milk composition. The *GHR* gene is implicated in lipid and carbohydrate metabolism and plays a pivotal role in the growth and development of mammary gland by initiating and maintaining lactation (Parmentier et al., 1999).

**Table 3** reports candidate genes located in 2.0 Mb SNP windows that explained the highest additive genetic variances for milk production under heat stress conditions. One genomic region on BTA15 (75.59–77.59 Mb) was strongly associated with the level of milk yield under heat stress across all three parities. This region harbors candidate genes such as *PEX16, MAPK8IP1, CREB3L1*, and *CRY2* that are all implicated in the cellular response to heat stress. Gene *MAPK8IP1* is involved in controlling cellular response to heat shock, which in turn increases transcription activity of several heat stress responsive genes that control several functions, including cell survival, cell proliferation, and apoptosis. Reactive oxygen species (ROS) are produced as a response to heat stress at the cellular level and these ROS cause cellular necrosis leading to cell death. Interestingly, gene *MAPK8IP1* is involved in suppressing heat stress induced ROS production and cellular apoptosis (Li et al., 2018). Gene *PEX16* is involved in cell membrane biosynthesis and plays an important role in cell protection against heat shock (Farr et al., 2016). Gene *CREB3L1* is implicated in endoplasmic reticulum stress response caused due to the accumulation of misfolded proteins and promotes cell survival during heat stress (Greenwood et al., 2015). Gene *CRY2* is involved in thermotolerance and the knockdown of *CRY2* increases sensitivity to temperature (Sanchez-Bermejo et al., 2015).

**Table 3:** Putative candidate genes located in 2.0 Mb SNP windows that explain the highest genetic variance for milk yield under heat stress conditions across the first three parities.

Chr.	Pos. (Mb)	Genetic Variance (%)			Candidate genes	Functions
		Par 1	Par 2	Par 3		
BTA5	96.9–98.9	–	–	0.85	CDKN1B DUSP16	removal of misfolded proteins, regulation of oxidative stress
BTA14	1.65–3.65	1.40	1.27	–	HSF1, EEF1D, VPS28, TONSL	molecular chaperone, promotion of cell survival under heat stress, DNA repair and maintenance of genome stability
BTA15	75.6–77.6	0.57	0.83	0.63	PEX16, MAPK8IP1, CREB3L1, CRY2	cellular response to stress, DNA replication, activation of heat stress target genes involved in cell survival, cell proliferation and apoptosis

Additionally, our whole-genome scans detected one genomic region located on BTA14 (1.65–3.65 Mb) that explained more than 0.5% of the additive genetic variance of milk yield under heat stress conditions for first and second parity. Notably, this region harbors genes *HSF1, EEF1D, VPS28, TONSL* which are also involved in cellular response to heat stress. Gene *HSF1,* upon heat stimulus, binds gene promoters containing heat shock elements and activates the expression of these genes that act as molecular chaperones and promote cell survival under heat stress conditions (Calderwood et al., 2010). Gene *EEF1D* regulates the expression of heat shock responsive genes through the association with heat shock transcription factors (Cui et al., 2016). Gene *VPS28* is a vacuolar protein sorting gene involved in heat shock resistance (Jarolim et al., 2013). Gene *TONSL* is involved in DNA repair and maintenance of genome stability in the presence of DNA damaging stimulus such as heat shock (Saredi et al., 2016).

Another genomic region on BTA5 (96.94–98.94 Mb) explained more than 0.5% of the additive genetic variance for milk yield under heat stress for the third parity. This region harbors candidate genes *CDKN1B* and *DUSP16.* Gene *CDKN1B* is an oxidative stress related gene which is upregulated during heat stress and is involved in apoptosis by selectively removing heat-induced protein aggregates, and hence reducing cellular proteotoxic stress (Logan and Somero, 2011). Gene *DUSP16* is another gene which is specifically induced during cellular heat stress. Indeed, gene *DUSP16* has a protective function against oxidative stress of cells during heat stress thereby promoting cell survival (Kapila et al., 2016).

Overall, our whole-genome scans have detected several genomic regions implicated in milk production level under heat stress conditions. Interestingly, these regions harbor candidate genes that are directly involved in the heat shock response, apoptosis, and oxidative stress.

### Gene-Set Analysis

Of the 58,046 SNP markers evaluated in the whole-genome association mapping, a total of 27,488 SNPs were located either within annotated genes or at most 15 kb upstream or downstream from annotated genes. This set of SNPs marked a total of 17,238 genes annotated in the UMD 3.1 bovine reference genome. A subset of 798 of these 17,238 genes were flagged by at least one relevant SNP (top 5% of the SNP effects distribution) in at least two parities, and hence, these 798 genes were defined as associated with milk production under heat stress conditions.

**Figure 2:**
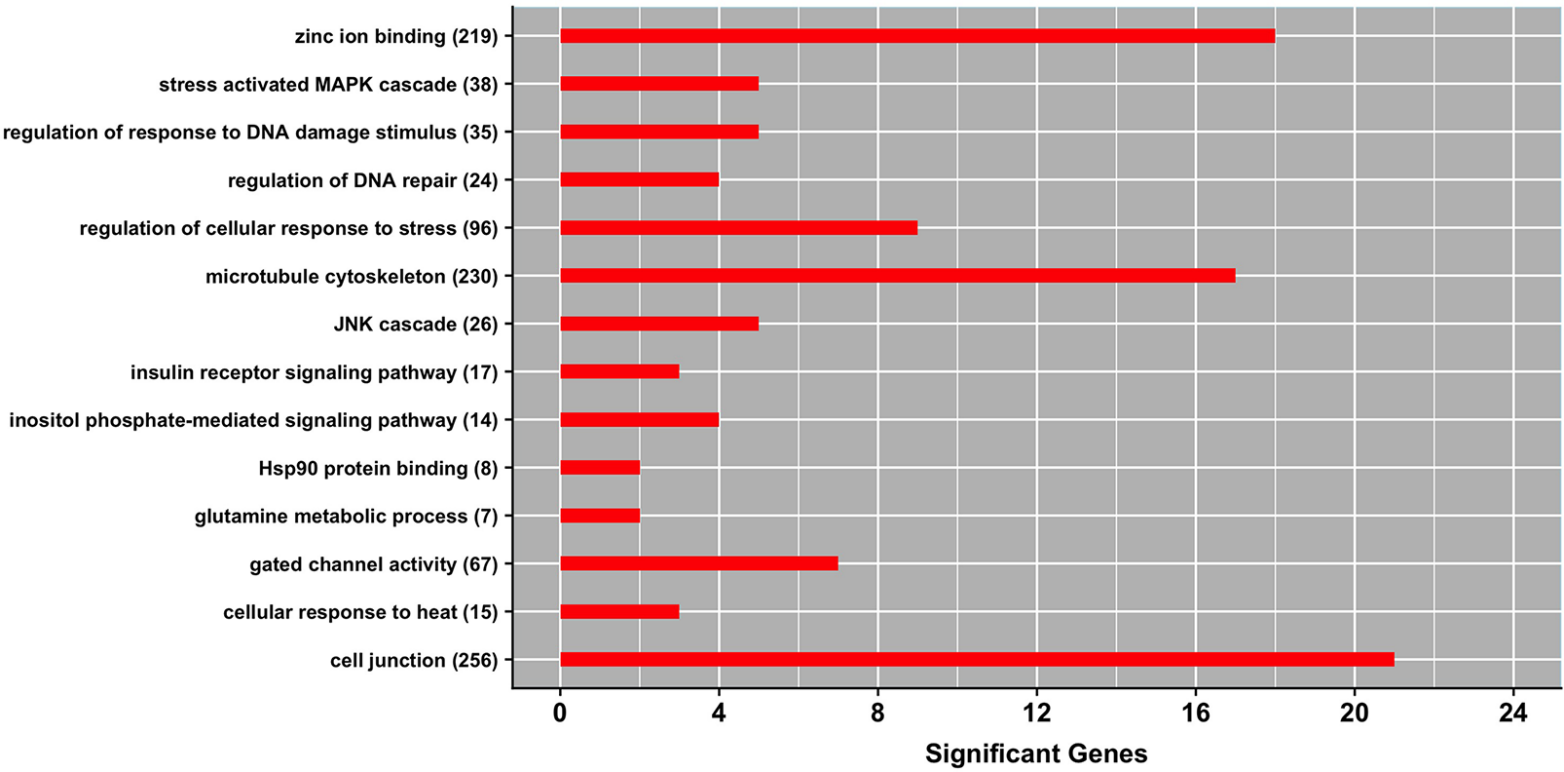
Gene ontology (GO) gene-set terms significantly enriched with genes associated with milk yield under heat stress conditions. The bars show the total number of genes associated with heat stress response per each significant term, and numbers within parenthesis show total number of genes in the GO term. The significance level was set at *P*-value ≤ 0.05 (Fisher’s exact test).

**Figure 2** shows a set of GO terms that were significantly enriched with genes affecting milk production under heat stress conditions. Several of these GO terms are related to the heat shock protein family. For instance, *cellular response to heat* (GO:0034605) and *regulation of cellular response to stress* (GO:0080135) are among the most significant gene-sets. These functional terms harbor several heat stress responsive genes, including *HSF1* which is directly implicated in cellular protection by maintaining proper protein folding and preserving cytoskeleton integrity during heat stress conditions. Notably, a recent transcriptome analysis revealed that the GO term *cellular response to stress* was significantly enriched with genes differentially expressed in blood of Holstein bull calves subjected to heat stress (Srikanth et al., 2017). Similarly, Li et al. (2015) reported GO term *response to temperature stimulus* being significantly enriched with genes differentially expressed in heat-treated bovine memory epithelial cells (BMECs) as a response to heat stress conditions. Hence, our study provides further evidence of a close relationship between thermotolerance, in this case measured as level of milk yield, and the stress response pathway.

Two other GO terms implicated in the level of milk production under heat stress conditions are *regulation of DNA repair* (GO:0006282) and *regulation of response to DNA damage stimulus* (GO:2001020). These functional gene-sets promote efficient DNA repair through recruitment of heat shock proteins and maintenance of genomic integrity during heat stress conditions. Interestingly, Collier et al. (2006) reported upregulation of genes associated with *DNA repair* when bovine mammary epithelial cells were exposed to heat stress.

The term *inositol phosphate mediated signaling pathway* (GO:0048016) was another GO term significantly enriched with genes implicated in maintaining milk production under heat stress. Notably, this gene-set harbors gene *ITPR2* which is directly implicated in sweating (Klar et al., 2014). Other significant GO terms include *insulin receptor signaling pathway* (GO:0008286), *JNK cascade* (GO:0007254), *stress activated MAPK cascade* (GO:0051403), and *glutamine metabolic process* (GO:0006541)*.* It is well-documented that heat stress causes the accumulation of reactive oxygen species in the cells, which in turns mediates MAPK activation pathway that is directly implicated in regulating apoptosis (Wada and Penninger, 2004).

Three GO terms classified into the molecular function domain showed an overrepresentation of genes associated with heat stress response including *Hsp90 protein binding* (GO:0051879), *zinc ion binding* (GO:0008270), and *gated channel activity* (GO:0022836). These pathways are closely related with sweat production, molecule and ion transport, and improving integrity of mammary epithelium under heat stress conditions. Additionally, there were two GO terms classified into cellular components domain that showed an overrepresentation of candidate genes, namely *cell junction* (GO:0030054) and *microtubule cytoskeleton* (GO:0015630)*.* Interestingly, Collier et al. (2006) reported upregulation of genes associated with *junctional adhesion* and *Hsp90-organizing protein* during the heat shock response in bovine mammary epithelial cells. These functional terms harbor genes that are important for maintaining the permeability of the cells, preventing the leakage of the cells, preserving cytoskeleton integrity and cell morphology during heat stress.

**Figure 3:**
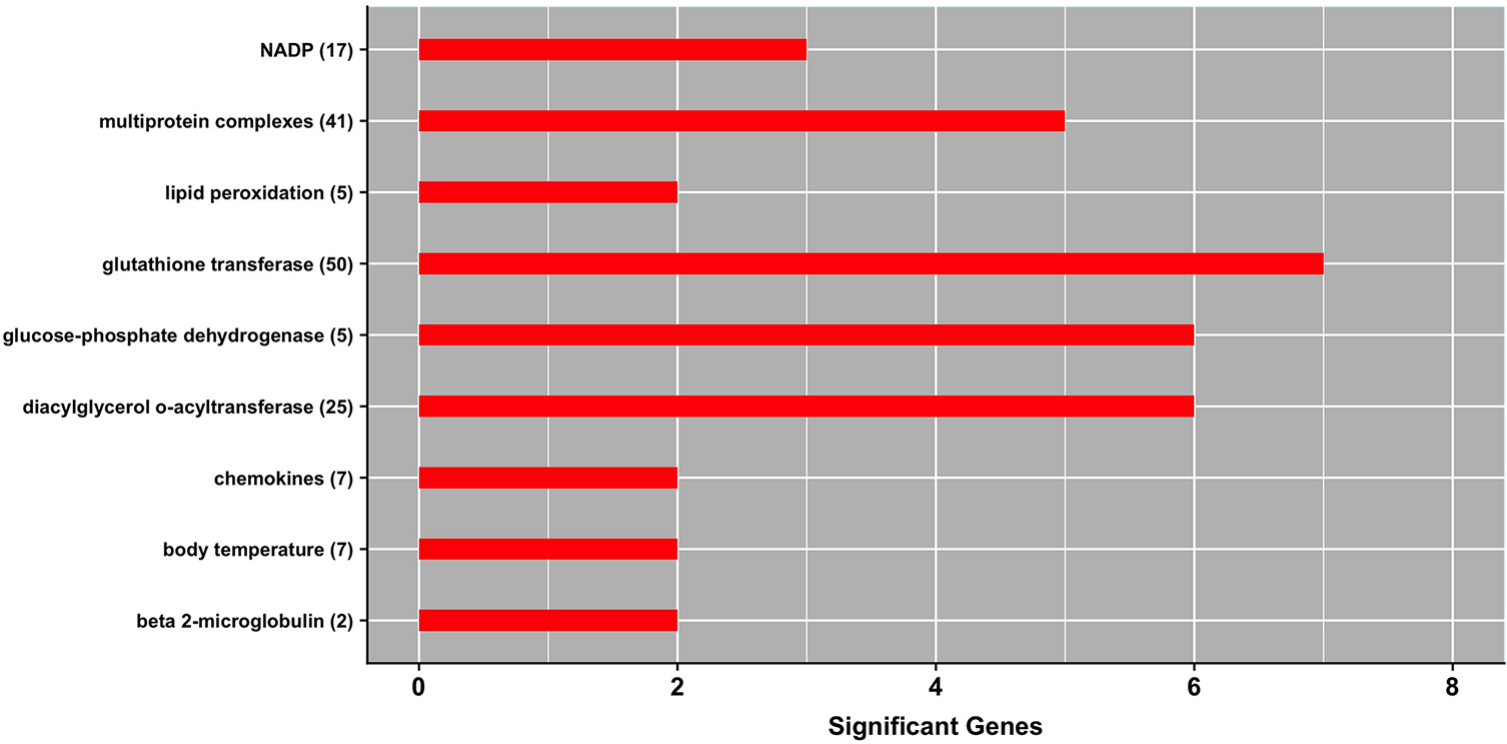
MeSH gene-set terms significantly enriched with genes associated with milk yield under heat stress conditions. The bars show the total number of genes associated with heat stress response per each significant term, and numbers within parenthesis show total number of genes in the MeSH term. The significance level was set at *P*-value ≤ 0.05 (Fisher’s exact test).

**Figure 3** shows a panel of MeSH terms that were enriched with genes underlying milk production under heat stress conditions. These significant MeSH terms include *body temperature* (D001831), *lipid peroxidation* (D015227), *multiprotein complexes* (D046912), and *chemokines* (D018925).

## Conclusions

Heritability estimates for milk yield, fat yield, and protein yield at heat stress conditions ranged between 0.13 to 0.31, while genetic correlations between general and thermotolerance additive genetic effects were negative and ranged from −0.18 to −0.68. Our findings reinforce the idea that there is a negative genetic relationship between production and thermotolerance. Our results also support the initial hypothesis that continued selection for milk volume and milk components without considering thermotolerance will result in greater susceptibility to heat stress. We also performed whole-genome scans and gene-set enrichment analyses with the purpose of identifying individual genes and functional gene-sets affecting milk production under heat stress conditions. We found that thermotolerance is a quantitative trait affected by several regions across the genome, with some prominent peaks on BTA5, BTA14, and BTA15. Moreover, the gene-set analysis revealed significant functional terms including the heat shock protein family, cellular response to stress, and response to DNA damage stimulus. Overall, this study contributes to a better understanding of the genetics underlying milk production under heat stress and points out novel opportunities for improving thermotolerance in dairy cattle.

## Data Availability Statement

The phenotypic and genotypic data analyzed in this study were obtained from North Florida Holsteins (Bell, FL), University of Florida Dairy Research Unit (Alachua, FL), Dairy Record Management System (Raleigh, NC), and Council on Dairy Cattle Breeding (Bowie, MD). These datasets were used under agreement, and hence, are not publicly available. However, data are available upon request to FP and with the permission of North Florida Holsteins, University of Florida Dairy Research Unit, and Cooperative Dairy DNA Repository.

## Author Contributions

FP and AS conceived and designed the study. AS conducted all analyses and drafted the manuscript. RA-A, IA, and FP assisted with the analysis and the interpretation of the results. All authors read and approved the manuscript before submission.

## Funding

This research was supported by funds from the Southeast Milk Inc. Milk Checkoff Program, and from the Florida Agricultural Experiment Station, University of Florida.

## Conflict of Interest

The authors declare that the research was conducted in the absence of any commercial or financial relationships that could be construed as a potential conflict of interest.
